# The Landmark Series: Neoadjuvant Therapy for Patients with Resectable Gastroesophageal Junction Adenocarcinomas

**DOI:** 10.1245/s10434-026-20114-4

**Published:** 2026-07-07

**Authors:** Morgan F. Pettigrew, Natalie G. Coburn, Yanghee Woo, Matthew R. Porembka

**Affiliations:** 1 Division of Surgical Oncology, Department of Surgery, University of Texas Southwestern Medical Center, Dallas, TX USA; 2 Division of Surgical Oncology, Department of Surgery, University of Toronto, Toronto, ON Canada; 3 Division of Surgical Oncology, Department of Surgery, City of Hope, Duarte, CA USA

## Abstract

Neoadjuvant therapy is a cornerstone of treatment for resectable gastroesophageal junction (GEJ) tumors. Despite substantial advancements, optimal regimen selection remains challenging owing to heterogeneity in clinical trial inclusion criteria and treatment algorithms. This review synthesizes findings from pivotal randomized trials to guide evidence-based selection of neoadjuvant treatment for GEJ adenocarcinoma. In 2006, the MAGIC trial established a survival benefit with perioperative chemotherapy with epirubicin, cisplatin, and fluorouracil (ECF) compared with surgery alone. Subsequently, in 2012, CROSS demonstrated improved overall survival (OS) and R0 resection rates with neoadjuvant chemoradiotherapy (CRT) using carboplatin and paclitaxel compared with surgery alone. CRT increased pathologic complete response (pCR) rates and decreased local recurrence rates, though it had limited impact on distant recurrence rates. In 2018, the FLOT4 trial demonstrated that perioperative FLOT (fluorouracil, leucovorin, oxaliplatin, docetaxel) improved overall survival and pCR rates compared with ECF, making FLOT the new standard for perioperative chemotherapy. Several trials have since compared perioperative chemotherapy with neoadjuvant CRT. Neo-AEGIS found no survival difference; however, the predominance of ECF rather than FLOT in the chemotherapy arm limits its pertinence in the modern era. TOPGEAR demonstrated improved pCR with the addition of preoperative radiotherapy to FLOT, but no survival advantage. More recently, ESOPEC established FLOT’s superiority over CROSS for OS, and MATTERHORN demonstrated improved event-free survival with the addition of immunotherapy to perioperative FLOT. FLOT is the current standard for neoadjuvant treatment in GEJ adenocarcinoma due to its superior survival outcomes. Integration of immunotherapy represents a promising avenue to further improve outcomes.

Neoadjuvant therapy is recommended for many resectable gastroesophageal junction (GEJ) tumors.^1^ The optimal neoadjuvant therapy regimen has been a focus of recent research, leading to significant advancements in multimodal treatment strategies. Foundational trials such as the MAGIC^2^ and CROSS^3^ established the survival benefit of multimodal therapy compared with surgery alone. More recent literature, including FLOT4,^4^ Neo-AEGIS,^5^ TOPGEAR,^6^ ESOPEC,^7^ and MATTERHORN,^8^ compares multimodal treatment strategies and provides key insights into the benefits and limitations of these strategies.

A key limitation of the available data is the lack of uniformity across clinical trials. Variability in inclusion criteria, tumor histology, location, and disease burden complicates direct comparisons between trial regimens, resulting in limited ability to define a single standard of care. Moreover, neoadjuvant regimens must balance efficacy with tolerability to ensure treatment completion and surgical eligibility. Key considerations when selecting a neoadjuvant treatment approach include whether the treatment achieves tumor downstaging, increases R0 resection rates, results in long-term disease control, and improves overall survival.

In this review, we aim to synthesize findings of pivotal randomized controlled trials to evaluate the efficacy of neoadjuvant treatment strategies for resectable GEJ adenocarcinoma. The results of these trials should provide a framework for determining optimal neoadjuvant treatment.

## Materials and Methods

### 2006 MAGIC^2^

#### Purpose/Rationale/Hypotheses

MAGIC investigated whether perioperative chemotherapy with epirubicin, cisplatin, and fluorouracil (ECF) could improve survival in patients with resectable gastric, gastroesophageal, or esophageal adenocarcinoma compared with surgery alone. The rationale was that preoperative chemotherapy may increase curative resections by downstaging tumors and treating micrometastases, while postoperative chemotherapy might eradicate residual disease. The hypothesis was that the addition of perioperative ECF would improve overall and progression-free survival (PFS) compared with surgery alone.

#### Study Design

MAGIC included patients with resectable adenocarcinoma of the stomach, GEJ, or lower esophagus. Patients were randomized to receive either perioperative chemotherapy with three cycles of ECF before and after surgery or surgery alone. The primary end point was overall survival (OS), with secondary end points including PFS, tumor downstaging, and surgical outcomes.

#### Results

The study cohort included 503 patients, with 250 patients in the perioperative chemotherapy arm and 253 in the surgery alone arm. Perioperative chemotherapy significantly improved survival outcomes. With a median follow-up of 4 years, the hazard ratio (HR) for death in the chemotherapy group was 0.75, 95% confidence interval (CI), 0.60–0.93 (*P* = 0.009). The 5-year OS was 36% in the chemotherapy group versus 23% in the surgery-alone group. The PFS HR was 0.66 (95% CI, 0.53–0.81; *P* < 0.001), indicating improved disease control. Tumor downstaging was more pronounced in the chemotherapy group, with a smaller median tumor size (3 cm versus 5 cm; *P* < 0.001), higher proportion of T1–T2 tumors (52% versus 37%; *P* = 0.002), and higher proportion of R0 resections (79% versus 70%; *P* = 0.03). Postoperative complication rates were approximately 45%, and 30-day mortality was approximately 6% for both groups. While 86% of patients completed all preoperative chemotherapy, only 42% of patients completed all six planned chemotherapy cycles.

#### Conclusions and Commentary

MAGIC established the survival benefit of perioperative chemotherapy and shifted the treatment paradigm for resectable gastroesophageal cancer from surgery alone to a multimodal approach; however, there were challenges in completing chemotherapy, particularly in the postoperative setting. Although the addition of preoperative therapy improved the amount of systemic therapy received, increasing receipt of postoperative chemotherapy remained a key area for improvement. Additionally, the trial design did not separate the impact of preoperative and postoperative chemotherapy on survival outcomes, limiting the ability to determine which component contributed most to the observed survival benefit. Lastly, the inclusion of GEJ tumors was not part of the original study design but was incorporated later; as a result, only 11% of enrolled patients had GEJ adenocarcinoma, limiting its applicability to this subgroup.

### 2012 CROSS^3^

#### Purpose/Rationale/Hypotheses

CROSS investigated whether neoadjuvant chemoradiotherapy (CRT) followed by surgery would improve OS compared with surgery alone in patients with potentially curable esophageal or GEJ cancer. The study was driven by the poor outcomes associated with esophageal and GEJ cancer, primarily owing to high rates of positive resection margins and recurrence. The hypothesis was that preoperative CRT would improve tumor downstaging, resection rates, and survival compared with surgery alone.

#### Study Design

This phase III multicenter randomized controlled trial included patients aged 18–75 with adenocarcinoma, squamous-cell carcinoma (SCC), or large-cell undifferentiated carcinoma of the esophagus or GEJ (clinical stages T1N1 or T2-3N0-1, M0). Eligible patients were stratified by histology, treatment center, nodal status as determined by endoscopic ultrasound, and performance status. The intervention group received neoadjuvant CRT consisting of five weekly doses of carboplatin and paclitaxel, along with 41.4 Gy in 23 fractions of radiotherapy, followed by surgery. The control group underwent surgery alone. The primary end point was OS. Secondary end points included disease-free survival (DFS), R0 resection, pCR, and postoperative morbidity and mortality. The study was powered to detect a difference in median OS (22 months for CRT versus 16 months for surgery alone).

#### Results

A total of 368 patients were analyzed, with 178 in the CRT group and 188 in the surgery-alone group. The majority (75%) had adenocarcinoma. After a median follow-up of 45 months, median OS was 49 months in the CRT group versus 24 months in the surgery-alone group (HR 0.66, 95% CI 0.50–0.87; *P* = 0.003). The 5-year OS rate was 47% in the CRT group versus 34% in the surgery-alone group, and the 10-year OS rate was 38% in the CRT group versus 25% in the surgery-alone group. Median DFS was not reached in the CRT group versus 24 months in the surgery-alone group. CRT significantly improved R0 resection rates (92% versus 69%; *P* < 0.001) and resulted in a 29% pCR rate. Postoperative complications, in-hospital mortality rate, and 30-day postoperative mortality were similar between groups.

#### Conclusions and Commentary

CROSS provided compelling evidence that neoadjuvant CRT improves OS, DFS, and R0 resection rates compared with surgery alone, without increasing perioperative morbidity or mortality. The study established neoadjuvant CRT as a cornerstone of treatment for esophageal and GEJ cancers, particularly for locally advanced tumors. However, CROSS did not include patients with T4 tumors, tumors larger than 5 cm, or N2 disease, limiting its applicability in patients with more advanced locoregional disease, especially as patients with clinically N0 disease benefited more than those with N1 disease. Additionally, the inclusion of multiple histologic subtypes further cautions against universal application of this regimen, particularly as the greatest benefit was observed in patients with SCC rather than adenocarcinoma. Further, although CROSS has a favorable effect on local response, it has no impact on distant recurrence rates, which were 27% in the CRT group and 28% in the surgery alone group.^9^

### 2019 FLOT4^4^

#### Purpose/Rationale/Hypotheses

FLOT4 compared perioperative chemotherapy using FLOT (fluorouracil, leucovorin, oxaliplatin, and docetaxel) with the standard ECF/ECX regimen in patients with resectable gastric or GEJ adenocarcinoma. Prior studies, such as MAGIC, established the role of perioperative chemotherapy for resectable tumors, but the efficacy of ECF was suboptimal, prompting the need for more effective regimens, such as FLOT, which had shown promising results in the metastatic setting. The hypothesis was that FLOT would improve OS compared with ECF/ECX without significantly increasing toxicity or surgical morbidity.

#### Study Design

This was a phase II/III, open-label, randomized trial conducted across 38 centers in Germany. Patients with locally advanced gastric or GEJ adenocarcinoma (cT2 or higher, cN+, or both) and no distant metastases were included. The study randomized patients to receive either FLOT (four cycles preoperatively and four cycles postoperatively) or ECF/ECX (three cycles preoperatively and three cycles postoperatively). Patients were stratified by performance status, age, nodal involvement, and tumor location (Siewert I versus II/III versus stomach). The primary end point was OS, and secondary end points included DFS, R0 resection rate, pathologic tumor response, surgical outcomes, and adverse events. The trial was powered to detect a hazard ratio (HR) of 0.76 for OS, allowing for a dropout rate of up to 24%.

#### Results

After a median follow-up of 43 months, the FLOT group demonstrated superior survival outcomes. Median OS was 50 months with FLOT versus 35 months with ECF/ECX (HR 0.77, 95% CI 0.63–0.94; *P* = 0.012). The 5-year OS rate was 45% with FLOT compared with 36% with ECF/ECX. Median DFS was 30 months with FLOT versus 18 months with ECF/ECX (HR 0.75, 95% CI 0.62–0.91; *P* = 0.004). Pathologic and surgical outcomes also favored FLOT, with higher R0 resection rates (85% versus 78%; *P* = 0.016) and a higher pCR rate (25% versus 15%; *P* = 0.0008).

Despite the increased intensity of FLOT, postoperative complication rates were similar between groups (approximately 50% in both). However, the FLOT group had a higher incidence of grade 3/4 neutropenia (51% versus 39%), infections (18% versus 9%), and diarrhea (10% versus 4%), whereas ECF/ECX was associated with higher rates of grade 3/4 nausea (16% versus 7%) and vomiting (8% versus 2%). The completion rate of all planned chemotherapy cycles was higher in the FLOT group (46%) compared with the completion rates for ECF (37%).

#### Conclusions and Commentary

FLOT was the first perioperative chemotherapy regimen to demonstrate superiority over the ECF/ECX standard in gastric and GEJ adenocarcinomas, and FLOT4 established FLOT as the preferred perioperative chemotherapy regimen for these cancers. This study built upon MAGIC by demonstrating that a more intensive regimen could yield substantial improvements in survival. However, questions remained regarding FLOT’s direct comparison to neoadjuvant CRT and whether managing its toxicity would affect real-world implementation.

### 2023 Neo-AEGIS^5^

#### Purpose/Rationale/Hypotheses

Neo-AEGIS aimed to directly compare perioperative chemotherapy (primarily ECF/ECX or FLOT) with neoadjuvant CRT using the CROSS protocol in patients with locally advanced adenocarcinoma of the esophagus and GEJ. Prior clinical trials had established the efficacy of both approaches, but no randomized study had directly compared them. The hypothesis was that neoadjuvant CRT would provide superior survival outcomes compared with perioperative chemotherapy.

#### Study Design

This was a phase III, open-label, randomized trial conducted across 24 European centers. Patients with clinical T2-3, N0-3, M0 adenocarcinoma of the esophagus or GEJ were randomly assigned to receive either perioperative chemotherapy (ECF/ECX before 2018, FLOT after 2018) or neoadjuvant CRT using the CROSS protocol (41.4 Gy with carboplatin and paclitaxel followed by surgery). The primary end point was OS. Secondary end points were DFS, pathologic response, R0 resection rate, treatment-related toxicity, and operative outcomes. The trial was initially powered for superiority of neoadjuvant CRT but was later modified to a noninferiority design with a 5% margin for perioperative chemotherapy. Planned accrual was set to 540, but the trial terminated early at 377 patients due to the coronavirus disease-19 (COVID-19) pandemic and interim futility analysis.

#### Results

After a median follow-up of 39 months, no significant difference in OS was observed between the two groups. Median OS was 48 months with perioperative chemotherapy versus 49 months with neoadjuvant CRT (HR 1.03, 95% CI 0.77–1.38; *P* = 0.82). The 3-year OS rates were similar (55% versus 57%). Median DFS was also comparable (32 months with perioperative chemotherapy versus 24 months with neoadjuvant CRT, HR 0.89, 95% CI 0.68–1.17; P = 0.41).

Despite the lack of survival difference, neoadjuvant CRT was associated with a significantly higher pCR rate (12% versus 4%; *P* = 0.02) and a greater proportion of R0 resections (96% versus 82%; *P* = 0.0003). Patients in the chemotherapy group were more likely to experience vomiting, diarrhea, neutropenia, peripheral neuropathy, and alopecia, while those in the CRT group experienced more odynophagia. Most toxicities were grade 1–2, but grade 3–4 neutropenia was more common with perioperative chemotherapy (27% versus 6%; *P* < 0.0001). In terms of surgical outcomes, 88% of the perioperative chemotherapy group underwent surgery compared to 94% in the CRT group. The 90-day postoperative mortality rate was similar between the two groups (2–3%).

#### Conclusions and Commentary

Neo-AEGIS sought to address a critical knowledge gap by providing a head-to-head comparison of two widely adopted curative-intent strategies for GEJ adenocarcinoma, perioperative chemotherapy, and neoadjuvant CRT. The trial demonstrated no significant difference in OS or DFS between the two approaches. Although neoadjuvant CRT achieved higher pCR and R0 resection rates, these advantages did not translate into a long-term survival benefit, raising important questions about CRT’s effectiveness in controlling systemic disease. These findings reinforced the ongoing clinical equipoise between the two approaches. However, a major limitation of the trial was that only 15% of patients in the perioperative chemotherapy group received FLOT. As a result, this trial may underestimate the potential benefit of modern chemotherapy compared with CRT. In the era of emerging systemic therapies, further studies were still warranted to determine the optimal neoadjuvant approach for GEJ adenocarcinoma.

### 2024 TOPGEAR^6^

#### Purpose/Rationale/Hypotheses

TOPGEAR sought to assess whether adding preoperative CRT to perioperative chemotherapy could improve OS in patients with resectable gastric or GEJ adenocarcinoma compared with perioperative chemotherapy alone. The rationale behind this study was that while perioperative chemotherapy improves survival outcomes, the potential benefits of preoperative CRT, including enhanced tumor downstaging and pCR, remained unexplored. The study hypothesized that preoperative CRT combined with perioperative chemotherapy would enhance OS and PFS compared with perioperative chemotherapy alone.

#### Study Design

This phase III, randomized, multicenter trial was conducted across 70 international sites and included resectable gastric or Siewert II or III GEJ adenocarcinoma (T3–T4, N any). Patients were randomized to receive either preoperative CRT with perioperative chemotherapy or perioperative chemotherapy. Preoperative CRT consisted of chemotherapy (two cycles of ECF/ECX or three cycles of FLOT), followed by CRT (45 Gy over 5 weeks with concurrent fluorouracil or capecitabine), and postoperative chemotherapy (three cycles of ECF/ECX or four cycles of FLOT). Perioperative chemotherapy consisted of either three cycles of ECF/ECX or four cycles of FLOT, both pre- and postoperatively.

The primary end point was OS. Secondary end points were PFS, pCR, tumor downstaging, R0 resection, and adverse events. This was powered to detect a 24% reduction in mortality with preoperative CRT. Planned accrual was 752 patients but was later revised to 570 patients due to slower enrollment and a higher-than-expected event rate.

#### Results

After a median follow-up of 67 months, median OS was 46 months with CRT versus 49 months with chemotherapy alone (HR 1.05, 95% CI 0.83–1.31). Similarly, median PFS was 31 months in the CRT group versus 32 months in the chemotherapy group (HR for progression or death 0.98, 95% CI 0.79–1.22). While no survival benefit was observed, preoperative CRT did lead to improved tumor downstaging with higher pCR rates (17% in CRT group versus 8% in chemotherapy-only group) and a higher proportion of major pathologic responses (50% with CRT versus 29% with chemotherapy alone). However, R0 resection rates were similar between groups (92% with CRT versus 88% with chemotherapy alone).

CRT resulted in higher rates of grade 3+ toxicities (66%) compared with chemotherapy alone (61%), with most adverse events being gastrointestinal and hematologic. Surgical complications were comparable between groups (18% with CRT versus 16% with chemotherapy alone), and 90-day postoperative mortality was low (1% with CRT versus 2% with chemotherapy alone). However, fewer patients in the CRT group completed postoperative chemotherapy (48%) compared with the chemotherapy-only group (59%), suggesting that CRT may impact the feasibility of adjuvant treatment completion.

#### Conclusions and Commentary

TOPGEAR demonstrated that adding preoperative CRT to perioperative chemotherapy improved pCR and tumor downstaging but did not improve survival outcomes. Both strategies had comparable safety profiles, demonstrating the feasibility of integrating radiotherapy in the preoperative setting, but the lack of survival advantage calls into question the clinical utility of CRT. This trial ultimately suggests no clear benefit of preoperative CRT for resectable gastric and GEJ cancers, reinforcing perioperative chemotherapy as the standard of care. These findings align with prior data from the CRITICS trial, which also failed to show a survival benefit with CRT in a perioperative chemotherapy setting, emphasizing limited survival impact in modern multimodal care.^10^ However, the inclusion of both ECF/ECX and FLOT regimens does limit direct conclusions about radiotherapy’s role with each regimen.

### 2025 ESOPEC^7,11,12^

#### Purpose/Rationale/Hypotheses

ESOPEC sought to compare the efficacy of perioperative FLOT chemotherapy with neoadjuvant CRT using the CROSS protocol for improving survival for patients with resectable esophageal and GEJ adenocarcinoma. Prior studies, such as CROSS and FLOT4, had demonstrated the survival benefit of both approaches, but no true head-to-head comparison had been made to establish which regimen would provide the greatest long-term benefit. The study hypothesized that perioperative FLOT provides superior OS compared with neoadjuvant CROSS in resectable esophageal and GEJ adenocarcinoma.

#### Study Design

This was a phase III, multicenter, randomized trial conducted across 25 sites in Germany that included patients with resectable esophageal and GEJ tumors staged as cT1N+, cT2-T4a N any, M0. Siewert I tumors were eligible, and Siewert II and III were eligible if there was esophageal infiltration. Patients were randomized to receive either FLOT or CROSS. The primary end point was OS. Secondary end points were pCR, R0 resection, treatment-related toxicity, and postoperative mortality. The trial was powered to detect an HR of 0.645, requiring 218 events at a one-sided significance level of 2.5%.

#### Results

A total of 438 patients were randomized (221 to FLOT, 217 to CROSS). After a median follow-up of 55 months, perioperative FLOT was found to be superior to CROSS in terms of OS. Median OS was 66 months with FLOT versus 37 months with CROSS (HR 0.70, 95% CI 0.53–0.92, *P* = 0.012). The 3-year OS was 57% with FLOT versus 51% with CROSS, and 5-year OS was 51% with FLOT versus 39% with CROSS. FLOT was also superior in terms of PFS, with a median of 38 months with FLOT versus 16 months with CROSS (HR 0.66, 95% CI 0.51–0.85, *P* = 0.001). Although FLOT had slightly higher pCR rates (17% with FLOT versus 10% with CROSS), R0 resection rates were similar (94% with FLOT versus 95% with CROSS). Lymph node negativity rates were also similar **(**51% with FLOT versus 55% with CROSS).

Grade ≥ 3 adverse events were higher with FLOT (58%) compared with CROSS (50%), with serious adverse events occurring in 47% of FLOT patients versus 42% of CROSS patients. The 90-day postoperative mortality was higher with CROSS (6%) compared with FLOT (3%). While 92% of patients in both groups underwent surgery, completion of all planned neoadjuvant therapy was higher in the FLOT group (87% versus 68%). In the FLOT arm, 63% of patients started adjuvant chemotherapy, and 53% completed adjuvant therapy, which was improved from historical completion rates (46% in FLOT4).

#### Conclusions and Commentary

ESOPEC provided the first true head-to-head comparison of perioperative FLOT versus CROSS. Perioperative FLOT significantly improved OS compared with neoadjuvant CROSS in resectable esophageal and GEJ adenocarcinoma, establishing perioperative FLOT as the preferred regimen. However, a lower-than-expected proportion of patients in the CROSS arm completed all planned neoadjuvant therapy, which may have attenuated the potential efficacy of CRT, but also raises concerns about treatment tolerability and adherence in real-world settings. Additionally, the study did not report a breakdown of GEJ versus esophageal tumors, which may affect the generalizability of results to GEJ tumors. Nonetheless, ESOPEC provides compelling evidence in favor of FLOT over CROSS for patients with resectable GEJ adenocarcinoma.

### 2025 MATTERHORN^8,13^

#### Purpose/Rationale/Hypotheses

MATTERHORN aimed to evaluate whether the addition of durvalumab, an anti-PD-L1 immune checkpoint inhibitor (ICI), to standard perioperative FLOT could improve event-free survival (EFS) in patients with resectable HER2-negative gastric or GEJ adenocarcinoma. The rationale for this combination stemmed from preclinical data, which demonstrate that docetaxel and 5-fluorouracil, components of the FLOT regimen, enhance T cell–mediated antitumor immunity.^14,15^ Additionally, combination ICI and chemotherapy strategies have demonstrated efficacy for the treatment of gastric and GEJ cancers in the metastatic setting.^16–18^ The central hypothesis was that adding durvalumab to perioperative FLOT would increase pCR and ultimately improve long-term survival by enhancing systemic disease control.

#### Study Design

This was a phase III, multinational, randomized, double-blind, placebo-controlled trial enrolling patients with stage II-IVa HER2-negative resectable gastric or GEJ adenocarcinoma. Patients were randomized 1:1 to receive durvalumab at a dose of 1500 mg or placebo every 4 weeks, plus FLOT for

four cycles (two cycles each of neoadjuvant and adjuvant therapy), followed by durvalumab or placebo every 4 weeks for 10 additional cycles. Patients were stratified before randomization by geographic region (Asia versus other regions). The primary end point was EFS, defined as disease progression, recurrence, or death. Secondary end points included OS, DFS, pCR, R0 resection rate, and receipt of surgery.

#### Results

The full analysis population included 958 patients: 474 received perioperative FLOT plus durvalumab and 474 received FLOT plus placebo. Among the 948 patients, 19% were from Asia, 53% were from Europe, 9% were from North America, and 19% were from South America. Approximately 32% of patients in each arm had GEJ tumors. Median follow-up was 31.5 months. Median EFS was not reached in the durvalumab group versus 32.8 months in the placebo group (HR: 0.71, 95% CI 0.58–0.86, *P* < 0.001). Median OS was not reached in the durvalumab group, compared with 47.2 months in the placebo group (HR 0.67, 95% CI 0.50–0.90, *P* = 0.03).

pCR rate was 19% in the durvalumab arm and 7% in the placebo arm (relative risk 2.69, 95% CI 1.86–3.90). R0 resection rates were 92% in both arms. Grade ≥ 3 treatment-related adverse events occurred in 72% of patients in the durvalumab arm versus 71% in the placebo arm. Immune-related adverse events were more frequent in the durvalumab arm (23% versus 7%). In the durvalumab arm, 97% completed neoadjuvant therapy, 87% underwent surgery, 76% initiated adjuvant FLOT, and 48% completed adjuvant FLOT. In the placebo arm, 95% completed neoadjuvant therapy, 84% underwent surgery, 74% initiated adjuvant FLOT, and 51% completed adjuvant FLOT.

#### Conclusions and Commentary

MATTERHORN is the first global, randomized phase 3 trial to show improved EFS with an immunotherapy-based regimen in resectable gastroesophageal junction cancers. The improvement in event-free survival and a nearly 3-fold improvement in pCR rates indicate enhanced antitumor activity from ICI therapy. While OS favored the durvalumab arm, the difference did not meet the prespecified threshold for statistical significance. This pattern of delayed OS benefit is consistent with the previously described kinetics of immunotherapy and may become statistically significant with longer follow-up.^19^ Additionally, patients with diffuse-type histology did not experience improvement in EFS. Overall, MATTERHORN supports the addition of ICI to perioperative FLOT; however, careful patient selection is key, as not all subgroups benefited from the addition of ICI (Table 1).

**Table 1:** Summary of landmark trials in neoadjuvant and perioperative therapy for resectable gastroesophageal junction adenocarcinoma

Category	MAGIC^2^	CROSS^3^	FLOT4^4^	Neo-AEGIS^5^	TOPGEAR^6^	ESOPEC^7^	MATTERHORN^8^
Study Design							
Study arms	Perioperative ECF versus Surgery Alone	Neoadjuvant CRT (CROSS) versus Surgery Alone	Perioperative FLOT versus Perioperative ECF/ECX	Perioperative Chemotherapy (MAGIC/FLOT) versus CROSS	Perioperative Chemotherapy (FLOT/ECF) versus Perioperative CRT	Perioperative FLOT versus CROSS	Perioperative FLOT + PD-L1 Inhibitor versus FLOT + Placebo
Recruitment period	1994–2002	2004–2011	2010–2015	2015–2019	2013–2020	2015–2021	2020–2024
Country	United Kingdom	Netherlands	Germany	Europe	10 countries	Germany	International
Number of sites	Multiple centers	8	38	24	70	25	156
Sample size	503	366	716	377	570	438	948
Disease site	Gastric (74%), Esophageal (15%), GEJ (12%)	Esophageal (72%), GEJ (24%), Not classified (4%)	Gastric (44%), GEJ (56%)	Esophageal (69%), GEJ (31%)	Gastric (65%), GEJ (35%)	Esophageal (46%), GEJ (33%), Not classified (21%)	Gastric (68%), GEJ (33%)
Tumor histology	Adenocarcinoma	Adenocarcinoma (75%), Squamous Cell Carcinoma (23%), Other (2%)	Adenocarcinoma	Adenocarcinoma	Adenocarcinoma	Adenocarcinoma	Adenocarcinoma
Stage	cT2–T4a, N0–N3, M0	cT1N1, cT2–3, N0–1, M0	cT2–T4a, N0–N3, M0	cT2–T4a, N+, M0	cT3–T4, N0–N+, M0	cT1N+, cT2–T4a, N any, M0	Stage II–IVa
Primary end point	Overall Survival	Overall Survival	Overall Survival	Overall Survival	Overall Survival	Overall Survival	EFS
Secondary end points	PFS R0 resection rate	PFS R0 resection rate pCR	PFS R0 resection rate pCR	DFS R0 resection rate pCR	PFS R0 resection rate pCR	PFS R0 resection rate pCR	OS R0 resection rate pCR
Median follow-up	4 years	4 years	5 years	3 years	3 years	5 years	32 months
Results							
Median OS	HR 0.75 (*P* = 0.009)	49 months (CRT) versus 24 months (surgery) HR 0.66 (*P* = 0.003)	50 months (FLOT) versus 35 months (ECF/ECX) HR 0.77 (*P* = 0.012)	48 months (Chemo) versus 49 months (CRT) HR 1.03 (*P* = 0.82)	49 months (Chemo) versus 46 months (CRT) HR 1.05 (*P* = 0.82)	66 months (FLOT) versus 37 months (CRT) HR 0.70 (*P* = 0.01)	Not reached (Durvalumab) versus 33 months (Placebo) HR 0.71 (*P* < 0.001)
3-year OS	–	–	–	55% (Chemo) vs 57% (CRT)	–	57% (FLOT) vs 51% (CROSS)	–
5-year OS	36% (ECF) versus 23% (surgery)	47% (CRT) versus 34% (surgery)	45% (FLOT) versus 36% (ECF/ECX)	–	–	51% (FLOT) versus 39% (CROSS)	–
PFS	HR 0.66 (*P* < 0.001)	not reached in the CRT group versus 24 months in the surgery alone group, HR 0.50 (*P* < 0.001)	30 months with FLOT versus 18 months with ECF/ECX, HR 0.75 (*P* = 0.004)	32.4 months (Chemo) versus 24.0 months (CRT), HR 0.89 (*P* = 0.41)	32 months (Chemo) versus 31 months (CRT), HR 0.98 (*P* = 0.77)	38 months (FLOT) versus 16 months (CROSS), HR 0.66 (*P* = 0.001)	EFS: Not reached (Durvalumab) versus 33 months (Placebo) HR 0.71 (*P* < 0.001)
R0 resection rate	Higher R0 resections with ECF	92% (CRT) versus 69% (surgery)	85% (FLOT) versus 78% (ECF/ECX)	82% (Chemo) versus 96% (CRT)	88% (Chemo) versus 92% (CRT)	94% (FLOT) versus 95% (CRT)	92% (Durvalumab) versus 92% (Placebo)
pCR	–	29% (CRT)	25% (FLOT) versus 15% (ECF/ECX)	4% (Chemo) versus 12% (CRT)	8% (Chemo) versus 17% (CRT)	17% (FLOT) versus 10% (CRT)	19% (Durvalumab) versus 7% (Placebo)

*CRT* chemoradiotherapy, *GEJ* gastroesophageal junction, *PFS* progression-free survival, *pCR* pathologic complete response, *DFS* disease-free survival, *OS* overall survival, *HR* hazard ratio, *EFS* event-free survival

## Discussion

The landscape of neoadjuvant therapy for resectable GEJ adenocarcinoma has evolved substantially over the past two decades. Contemporary evidence establishes perioperative FLOT as the preferred neoadjuvant chemotherapy backbone for GEJ adenocarcinoma, with growing evidence supporting the addition of immunotherapy in select subgroups. FLOT is both safe and tolerable. In the FLOT4, ESOPEC, and MATTERHORN trials, 90%, 87%, and over 95% of patients, respectively, completed all planned neoadjuvant therapy. Moreover, completion rates for the entire perioperative regimen have improved over time, rising from 46% in FLOT4 to 53% in ESOPEC and 50% in MATTERHORN, suggesting advances in toxicity management have enhanced delivery of systemic therapy. Importantly, FLOT does not compromise the ability to proceed to surgery, as demonstrated by high rates of resection in FLOT4 (94%), ESOPEC (86%), and MATTERHORN (86%). FLOT provides effective local control by downstaging tumors and increasing the likelihood of curative resection. In FLOT4, 25% of tumors were ypT0-1, 49% achieved ypN0 status, and 85% received R0 resection. Similarly, ESOPEC demonstrated comparable R0 resection rates with both CROSS and FLOT, and MATTERHORN reported R0 resection rates exceeding 90% with FLOT alone, reinforcing its effectiveness in achieving complete tumor removal.

Survival outcomes further support the superiority of FLOT over other chemotherapy and CRT regimens. In FLOT4, the median OS for patients receiving FLOT was 50 months, compared with 35 months with ECF/ECX. In ESOPEC, FLOT led to an even longer median OS of 66 months, compared with 37 months in the CROSS arm. While CRT is associated with higher pCR rates, this advantage consistently fails to translate into improved long-term survival. ESOPEC provided the first definitive head-to-head evidence favoring perioperative FLOT over CROSS for GEJ adenocarcinoma, firmly establishing FLOT as the preferred regimen.

The MATTERHORN trial marks another pivotal advancement by incorporating immune checkpoint inhibition into the neoadjuvant setting. There is a compelling biologic rationale for neoadjuvant immunotherapy, as a greater in situ tumor burden before resection, and thus more available tumor antigens, may elicit a more robust immune response, potentially providing a more durable antitumor effect. The addition of durvalumab to perioperative FLOT significantly improved EFS and nearly tripled pCR rates without compromising surgical feasibility. MATTERHORN distinguishes perioperative ICI-based regimens from adjuvant-only approaches such as ATTRACTION-5, which failed to improve survival outcomes.^20^ While the KEYNOTE-585 trial, which evaluated perioperative pembrolizumab with cisplatin-based perioperative chemotherapy, showed improved pCR rates, it did not demonstrate a survival benefit.^21^ These results suggest that the choice of chemotherapy backbone, particularly the use of FLOT, may be critical to maximizing the efficacy of ICI therapy in this setting.

## Caveats and Limitations

Despite these data, direct comparisons between clinical trials remain challenging due to heterogeneous inclusion criteria, particularly differences in histology, tumor location, and disease burden. For example, the MAGIC trial included gastric, GEJ, and esophageal tumors but did not analyze GEJ adenocarcinomas separately, limiting its applicability to this subgroup. Similarly, while CROSS demonstrated a significant improvement in R0 resection rates, its population was a mix of adenocarcinoma and SCC, and greater benefit was observed in SCC than in adenocarcinoma. Additionally, FLOT4 established FLOT as the preferred chemotherapy regimen, but offered no direct comparison with CRT. More recent trials, such as Neo-AEGIS and ESOPEC, attempted direct comparisons, yet their findings were limited by heterogeneous treatment approaches and evolving surgical and systemic therapy standards.

Furthermore, despite improved treatment adherence in ESOPEC, where 53% of patients completed all planned chemotherapy, 5-year OS remains only 51%, indicating that there is still room for vast improvements in systemic therapy strategies and a critical need for novel treatment approaches to further enhance long-term survival outcomes. While ICI therapy may provide an avenue forward, OS data from MATTERHORN are not yet mature, limiting comparisons with previous trials. Additionally, the benefit of ICI therapy was not observed across all subgroups, including patients with PD-L1 expression < 1% and those with diffuse-type histology. These findings underscore the need for validated predictive biomarkers and careful patient selection to further optimize outcomes.

## Outstanding Questions

### Clarifying the Role of CRT

Although perioperative FLOT is now the preferred neoadjuvant backbone for most patients with resectable GEJ adenocarcinoma, the role of CRT remains incompletely defined. CROSS established that neoadjuvant CRT improves local disease control, R0 resection rates, and pCR compared with surgery alone; however, its limited effect on distant recurrence suggests that its systemic therapy component may be insufficient for patients at high risk of occult metastatic disease.

Several strategies have been proposed to address this limitation, including adjuvant systemic therapy after CRT,^9^ integration of CRT into perioperative chemotherapy,^22^ and combination of CRT with ICI.^23,24^ CheckMate 577 supports adjuvant nivolumab for patients with residual disease after neoadjuvant CRT and surgery,^23^ but whether CRT followed by adjuvant systemic therapy can outperform perioperative FLOT remains unknown. Similarly, TOPGEAR^6^ showed that adding preoperative CRT to perioperative chemotherapy improved pathologic response but did not improve survival, limiting support for routine CRT integration. Radiation may also enhance antitumor immunity, but the clinical value of combining CRT with ICI therapy remains investigational.^24–26^ Therefore, CRT may be best reserved for selected patients at high risk of locoregional failure, while future studies should define its role in response-adapted and biomarker-directed treatment strategies.

### Molecular Marker-Based Neoadjuvant Strategies

As systemic therapy evolves, treatment selection for resectable GEJ adenocarcinoma will increasingly depend on molecular subtype. Mismatch repair-deficient and microsatellite instability-high (dMMR/MSI-H) tumors, which represent approximately 5–10%^27,28^ of GEJ adenocarcinomas, appear to derive limited benefit from conventional perioperative chemotherapy but demonstrate marked sensitivity to ICI therapy.^29–31^ In NEONIPIGA, neoadjuvant nivolumab plus ipilimumab achieved a pCR rate of 59% among patients with dMMR/MSI-H gastric or GEJ adenocarcinoma who underwent resection.^30^ Similarly, INFINITY demonstrated a pCR rate of 60% and a major pathologic response rate of 80% with neoadjuvant tremelimumab plus durvalumab in patients with resectable dMMR/MSI-H gastric or GEJ adenocarcinoma.^31^ These findings indicate that selected patients with dMMR/MSI-H disease may be candidates for ICI-based neoadjuvant strategies without traditional cytotoxic chemotherapy.

HER2-positive GEJ adenocarcinoma represents another clinically relevant subgroup. Although HER2-directed therapy is established in metastatic disease,^32^ its role in the perioperative setting remains undefined. Phase II studies such as HERFLOT^33^ and PETRARCA^34^ suggest that adding HER2-directed therapy to perioperative chemotherapy can improve pCR rates, but larger randomized data have not yet demonstrated a clear survival benefit.^35^ Moreover, emerging combinations of chemotherapy, HER2-directed therapy, and immune checkpoint inhibition have reported encouraging pCR rates, but these remain investigational.^36^ Importantly, MATTERHORN^8,13^ excluded HER2-positive disease, leaving this subgroup without a clearly defined immunotherapy-based perioperative standard. Future trials should incorporate molecular stratification, evaluate survival rather than pathologic response alone, and determine whether dMMR/MSI-H and HER2-positive tumors warrant distinct neoadjuvant strategies.

### Role of Organ Preservation with Active Surveillance in Patients who Achieve a Complete Clinical Response

Another emerging area of interest is the potential role of active surveillance in patients who achieve a complete clinical response (cCR) following neoadjuvant treatment. In rectal cancer, nonoperative management is increasingly considered in select patients who demonstrate a sustained cCR following neoadjuvant therapy, ^37,38^ leading researchers to explore whether a similar approach could be applied to esophageal and GEJ cancers. For patients who achieve cCR after neoadjuvant therapy, avoiding surgery could spare them the morbidity associated with esophagectomy, particularly in cases where local disease control is effectively achieved. However, data supporting this approach in GEJ adenocarcinoma remain limited, with one clinical trial ongoing,^39^ and further studies are required to determine which patients can safely undergo surveillance rather than resection.

## Conclusions

The available evidence strongly supports perioperative FLOT chemotherapy as the preferred neoadjuvant backbone for resectable GEJ adenocarcinoma, given its superior survival outcomes and systemic control, although combination regimens with ICI therapy are emerging as a new standard. While neoadjuvant CRT with CROSS remains an option for selected patients, it is less effective than FLOT. Significant questions remain regarding how best to optimize multimodal treatment strategies. Future trials should focus on refining patient selection, integrating novel systemic therapies, and identifying biomarkers to further individualize treatment approaches. By addressing these challenges, we can continue to improve survival and quality of life for patients with GEJ adenocarcinoma.
